# Weekend Effect on Mortality, Access to Renal Replacement Therapy, and Other Outcomes Among Patients With End-Stage Renal Disease: A Retrospective Analysis of the Nationwide Inpatient Sample

**DOI:** 10.7759/cureus.34139

**Published:** 2023-01-24

**Authors:** Fidelis E Uwumiro, Victory O Okpujie, Aminnah Oyesomi, Festa C Madu, Ayodeji Ilelaboye, Muhammed L Shielu, Ruth C Otu, Grace D Ogunkoya, Loveth S Ezennaya, Michael M Bojerenu

**Affiliations:** 1 Family Medicine, Our Lady of Apostles Hospital, Akwanga, NGA; 2 Internal Medicine, University of Benin Teaching Hospital, Benin City, NGA; 3 Internal Medicine, Sudan International University, Khartoum, SDN; 4 Internal Medicine, Nnamdi Azikiwe University, Awka, NGA; 5 Internal Medicine, Ladoke Akintola University of Technology, Ogbomosho, NGA; 6 Internal Medicine, Al Qunfudah General Hospital, Al Qunfudhah, SAU; 7 Internal Medicine, College of Health Sciences, Afe Babalola University, Ado Ekiti, NGA; 8 Internal Medicine, Lagos State University College of Medicine, Lagos, NGA; 9 Internal Medicine, St. Barnabas Hospital (SBH) Health System, New York, USA

**Keywords:** end-stage renal disease, nationwide inpatient sample, nationwide inpatient sample (nis), dialysis, renal replacement therapy, esrd, weekend effect

## Abstract

Background: A large body of research has been conducted on the "weekend effect," which is the reportedly increased risk of adverse outcomes for patients admitted to the hospital on weekends versus those admitted on weekdays. This effect has been researched in numerous patient populations, including sub-populations of end-stage renal disease (ESRD) patients, with varying conclusions.

Objectives: To assess whether differences in in-hospital mortality, access to renal replacement therapy (RRT), time to RRT, and other important outcomes exist in patients with ESRD or patients on RRT admitted on the weekend versus weekdays.

Design and setting: A retrospective cohort study was conducted using the 2018 Nationwide Inpatient Sample. Patients were included if they were adults with a principal or secondary diagnosis of ESRD or if they were admitted with a diagnosis related to initiation, maintenance, or complications of RRT. Patients admitted between midnight Friday and midnight Sunday were classified as weekend admissions. Primary outcome measurements included in-hospital mortality, in-hospital dialysis (peritoneal dialysis, hemodialysis, and continuous RRT), and renal transplantation (TP). Secondary outcomes included length of hospital stay (LOS) and total hospitalization charges.

Results: The study included 1,144,385 patients who satisfied the inclusion criteria. Compared with patients admitted on weekdays, patients with ESRD admitted on weekends had 8% higher adjusted odds of in-hospital mortality (OR: 1.08; 95% CI: 1.03-1.13; p = 0.002), 9% lower adjusted OR of any RRT over the weekend than on weekdays (OR: 0.91; 95% CI: 0.89-0.93; p = 0.000), lower RRT rates (within 24 hours) (adjusted OR: 0.71; 95% CI: 0.70-0.73; p = 0.000), higher odds of renal TP (adjusted OR: 1.32; 95% CI: 1.20-1.45; p = 0.000), and higher hospitalization charges (mean adjusted increase: $1451; p = 0.07).

Limitations: The limitations of the study include the use of retrospective data and an administrative database.

Conclusion: Compared with weekday admissions, patients with ESRD admitted on weekends had higher odds of mortality, higher mean hospitalization charges, and higher odds of renal TP. They had lower overall RRT rates, and a longer time to first RRT. However, the average LOS was similar for both weekend and weekday admissions.

## Introduction

The fifth and last stage of chronic kidney disease is end-stage renal disease (ESRD). Indications of ESRD include a glomerular filtration rate of less than 15 mL/min/1.73 m2, the requirement for dialysis or renal transplantation (TP), or death due to renal failure [1]. The crude incidence rate of ESRD in the US has risen since 2011, while the age-sex-race-standardized incidence rate appears to have plateaued since 2018. The number of people with ESRD peaked in 2019 at 808,330, an increase of 107% over pre-pandemic levels, and then declined slightly to 807,920 in 2020 [2]. Hemodialysis (HD) is the most extensively available renal replacement therapy (RRT) in the world, but it also has the greatest mortality rate, followed by renal TP and peritoneal dialysis (PD) [3].

The higher mortality associated with weekend patient admissions is a problem that has attracted scientific, political, and media interest on a global scale. It has been proposed that the weekend effect is caused by the failure of healthcare management organizations to improve care processes, such as providing access to specialists and life-saving diagnostic and treatment procedures 24 hours a day, seven days a week [4]. Theoretically, outcomes are expected to be worse for patients admitted on weekends compared to those admitted on weekdays because most renal transplants are performed on weekdays and many dialysis suites are not open throughout the weekend, resulting in a delay in RRT performance. Recent research suggests that the effect of weekend admission on mortality is limited to critical illnesses, particularly those requiring end-of-life care [5].

Diverse subpopulations of patients have been researched for the influence of weekend admission on mortality, with results indicating worse outcomes among patients admitted over the weekend. However, there is insufficient evidence associating weekend admissions with an increase in ESRD mortality. The purpose of this study was to determine whether there were variations in in-hospital mortality, access to RRT, and other significant outcomes between patients with ESRD hospitalized on the weekend versus weekdays using a large, population-based database in the United States and the International Classification of Diseases, Tenth Revision, Clinical Modification/Procedure Coding System (ICD-10-CM/PCS).

## Materials and methods

Design and data source

A retrospective cohort study was conducted using the 2018 Nationwide Inpatient Sample (NIS). The NIS is the largest all-payer inpatient care database accessible to the public in the US, with information on more than 35 million hospitalizations. It is developed and maintained by the Agency for Healthcare Research and Quality. Its large sample size is appropriate for generating national and regional estimates and facilitates analyses of rare diseases, uncommon procedures, and specific patient populations. The NIS records all hospitalizations in 48 states plus the District of Columbia and thus represents 97% of the US population [6,7]. The NIS uses a 20% stratified sample of discharges from community hospitals in the US, excluding rehabilitation and long-term acute care facilities. The self-weighting design of the NIS reduces the margin of error for estimates and delivers more stable and precise estimates than previous versions of the NIS [8]. Relevant patient sub-populations were identified by querying the 2018 database with the ICD-10-CM/PCS codes. Only precise and billable ICD-10-CM/PCS codes were utilized, avoiding all category, non-billable, and non-specific codes.

Study population

We queried the NIS 2018 database for persons aged 18 years or older with a primary or secondary diagnosis of ESRD. Patients were excluded if they were under the age of 18 years. This cohort was further separated into workday admissions (those admitted Monday through Friday) and weekend admissions (those admitted between midnight Friday and midnight Sunday). The subpopulations of the main cohort who had dialysis (HD or PD), continuous renal replacement therapy (CRRT), and TP were derived using a combination of statistical commands and the applicable ICD-10-CM/PCS codes. These codes are available in the Appendix.

Study variables

The length of hospital stay (LOS) and overall hospitalization costs are documented in the NIS database. HD, PD, and TP were identified using ICD-10-CM/PCS codes (Appendix). Weekend admissions were defined as those that took place on Saturday and Sunday, as defined by the weekend variable in the NIS. This classification has been used in other studies on ESRD and RRT [9-11]. The NIS records the number of days between a patient's admission and a procedure. Therefore, if the time to the procedure was indicated as zero or one day, dialysis or transplantation was considered to have been performed within 24 hours of admission.

Main outcome measurements

The primary outcome was in-hospital mortality among patients admitted on the weekend as compared to the weekdays. Secondary outcomes were overall RRT rates, access to early RRT (within 24 hours of admission), mean LOS, access to TP, and mean hospital charges for weekend and weekday admissions. Mean LOS, access to TP, and mean hospital charges were also compared between patients who had early RRT and those who did not.

Statistical analysis

Version 17 of Stata® (StataCorp LLC, College Station, Texas) was used to analyze the data. The software facilitates analysis to produce nationally representative and unbiased results, variance estimates, and p-values. All analyses were conducted using weighted samples for national estimates, in accordance with the Healthcare Cost and Utilization Project's specifications for using the NIS database. A thorough literature search was undertaken to identify potential confounders (age, sex, race, income in the patient's ZIP code, insurance type, hospital location/teaching status, hospital bed size, hospital region, and Charlson Comorbidity Index (CCI)), which were used in a univariable regression analysis to calculate unadjusted odds ratios (ORs) for the primary and secondary outcomes. Subsequently, a multivariate regression model was built by using only variables that were associated with the outcome of interest on univariable regression analysis at p < 0.2. Proportions were compared by using Fisher's exact test, and continuous variables were compared by using Student's t-test. All p-values were two-sided, and the threshold for statistical significance was set at 0.05.

Ethical considerations

Potential patient identifiers have been omitted from the NIS database. Since 2012, the NIS has also deleted state and hospital identifiers. This has increased patient anonymity and safety. Although considered and pursued, this research required no approval from an institutional review board.

## Results

Patient characteristics

**Figure 1:**
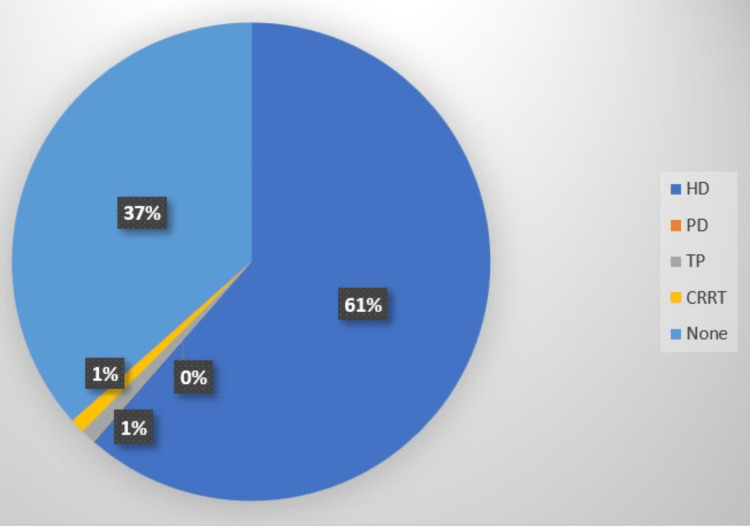
Proportion of patients receiving renal replacement therapy in the study population HD: hemodialysis; PD: peritoneal dialysis; TP: kidney transplantation; CRRT: continuous renal replacement therapy; None: not receiving any of the renal replacement therapies.

**Figure 2:**
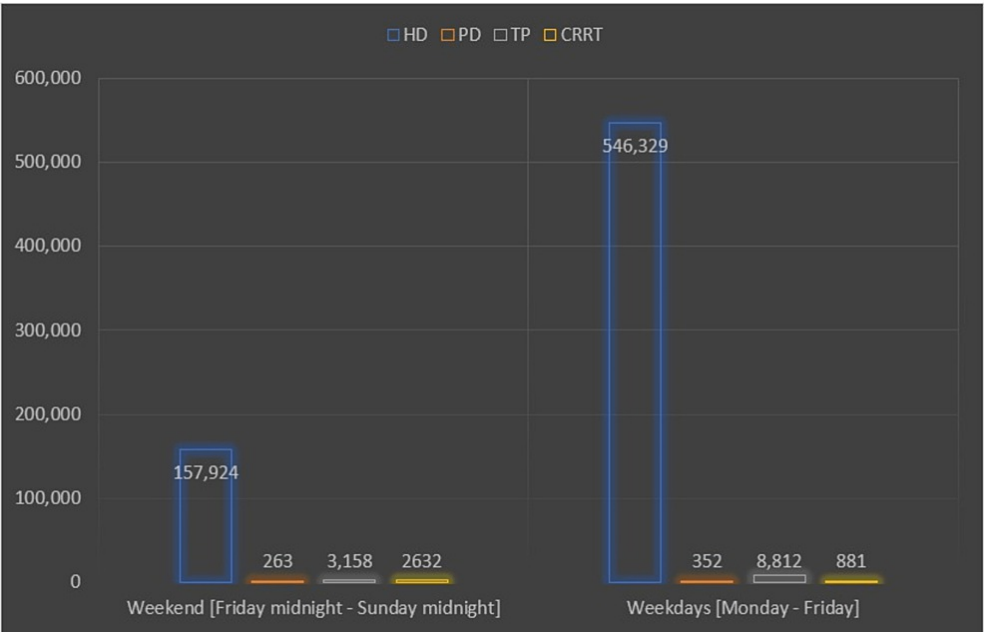
Number of patients who received renal replacement therapy on weekends versus weekdays HD: hemodialysis; PD: peritoneal dialysis; TP: kidney transplantation; CRRT: continuous renal replacement therapy.

The NIS 2018 database contained 35,521,883 million hospital discharges, of which 1,144,385 (3.2%) were studied. These patients were adults who had a primary or secondary discharge diagnosis of ESRD or whose admissions (as defined by ICD-10-CM/PCS codes) were directly related to the initiation of RRT or issues that occurred after starting RRT. Of the study population, 263,208 (23%) were admitted over the weekend compared with 881,176 (77%) admitted on weekdays. Table 1 and Figures 1, 2 summarize the demographic characteristics of the study.

**Table 1:** Patient and hospital characteristics HMO: health management organization; RRT: renal replacement therapy.

	Weekend admission	Weekday admission	P-value
Patient characteristics			
Number (%) of patients	263,208 (23%)	881,176 (77%)	
Women, No. (%)	121,075 (46%)	120,812 (46%)	0.80
Race/ethnicity, No. (%)			0.80
White	110,547 (42%)	370,093 (42%)	
Black	84,226 (32%)	290,788 (33%)	
Hispanic	44,745 (17%)	149,799 (17%)	
Asian or Pacific Islander	10,528 (4%)	35,247 (4%)	
Native American	2,632 (1%)	8,811 (1%)	
Other	7,896 (3%)	26,435 (3%)	
Mean age (in years)	61.8	62.1	
Charlson Comorbidity Index score			0.05
0	1,052 (.4%)	4,405 (0.5%)	
1	789 (.3%)	3,524 (0.4%)	
2	26,320 (10%)	87,236 (10%)	
≥3	234, 255 (89%)	784,246 (89%)	
Median annual income in patient’s zip code, US$, No. (%)			0.10
1-45,999	97,387 (37%)	326,035 (37%)	
46,000-58,999	68,434 (26%)	22,9105 (26%)	
59,000-78,999	55,273 (21%)	185,046 (21%)	
≥79,000	39,481 (15%)	123,364 (14%)	
Insurance type, No. (%)			0.70
Medicaid	197,406 (75%)	652,070 (74%)	
Medicare	34,217 (13%)	105,741 (12%)	
Private including HMO	28,952 (11%)	96,929 (11%)	
Uninsured	5,264 (2%)	17,623 (2%)	
RRT < 24 hours after admission, No. (%)	94,754 (36%)	387,717 (44%)	<0.001
Hospital region, No. (%)			<0.001
Northeast	42,113 (16%)	149,799 (17%)	
Midwest	52,641 (20%)	185,046 (21%)	
South	113,179 (43%)	370,093 (42%)	
West	55,273 (21%)	176,235 (20%)	
Hospital bed size, No. (%)			0.16
Small	44,745 (17%)	140,988 (16%)	
Medium	76,330 (29%)	264,352 (30%)	
Large	142,132 (54%)	475,835 (54%)	
Hospital location/teaching status, No. (%)			0.85
Rural	13,160 (5%)	44,058 (5%)	
Urban non-teaching	50,009 (19%)	167,423 (19%)	
Urban teaching	200,038 (76%)	669,693 (76%)	

In-hospital mortality based on the day of admission

The total in-hospital mortality rate in the study population was 4.6%. There were 53,545 recorded deaths. Compared to patients admitted on weekdays, patients admitted over the weekend had 8% higher odds of mortality (adjusted OR: 1.08; 95% CI: 1.03-1.13; p = 0.002) while keeping other confounders, such as age, sex, race, CCI, median income in patient’s ZIP code, hospital location/teaching status, hospital bed size, and hospital region, constant (Table 2). Similar results were obtained from univariable regression analysis (OR: 1.08; 95% CI: 1.03-1.13; p = 0.001). Patients with higher CCI scores also had increased odds of in-hospital mortality (adjusted OR: 1.18; 95% CI: 1.17-1.19; p = 0.00).

**Table 2:** Adjusted odds of in-hospital mortality among patients admitted over the weekend

Variables	Coefficient	Linearized standard error	t-value	P-value	95% CI (lower limit)	95% CI (upper limit)
Died						
Weekend admission	1.078	0.025	3.17	<0.001	1.029	1.129
Age (in years)	1.031	0.001	35.83	<0.001	1.029	1.032
Female	0.933	0.019	-3.37	0.001	0.897	0.971
Race (White as reference)	1					
Black	0.821	0.023	-7.14	<0.001	0.778	0.867
Hispanic	0.752	0.027	-7.81	<0.001	0.701	0.808
Asian or Pacific Islander	0.795	0.041	-4.40	<0.001	0.718	0.881
Native American	0.886	0.097	-1.10	0.27	0.715	1.099
Other	0.97	0.058	-0.52	0.61	0.863	1.090
Median household income for patients' ZIP code, US$ (1-45,999 as reference)	1					
46,000-58,999	0.944	0.026	-2.10	0.04	0.894	0.996
59,000-78,999	0.935	0.027	-2.31	0.02	0.883	0.990
79,000 or more	0.977	0.032	-0.70	0.48	0.915	1.043
Charlson Comorbidity Index	1.179	0.006	30.63	<0.001	1.166	1.191
Hospital region (Northeast as reference)	1					
Midwest	0.931	0.036	-1.85	0.07	0.863	1.004
South	1.059	0.038	1.60	0.11	0.987	1.136
West	1.146	0.045	3.49	<0.001	1.061	1.237
Hospital location/teaching status	1.123	0.025	5.15	<0.001	1.074	1.174
Hospital bed size (small as reference)	1					
Medium	1.108	0.042	2.71	0.01	1.029	1.193
Large	1.194	0.042	5.09	<0.001	1.115	1.278

In-hospital RRT rates and time to RRT based on the day of admission

During 62% of all admissions, at least one RRT (HD, CRRT, PD, or TP) was performed. Of the patients hospitalized on weekends, 61% received RRT, compared to 63% of those admitted on weekdays. The adjusted OR of any RRT over the weekend was 9% lower than on weekdays (OR: 0.91; 95% CI: 0.89-0.93; p = 0.000). Higher overall odds of RRT on the weekend were independently linked with Black and Hispanic race, as well as higher CCI scores.

In the studied population, the overall rate of PD was 0.001% (263). After controlling for patient and hospital-level variables, patients admitted on weekends had a higher chance of receiving PD than those admitted on weekdays (OR: 1.75; 95% CI: 1.20-2.57; p = 0.000). Admission to an urban teaching hospital, a large hospital, and the Northeast and Midwest areas were all independently related to a higher likelihood of PD over the weekend. Female sex and Asian or Pacific Islander race had no effect on the probability of PD (Table 3).

**Table 3:** Adjusted odds of peritoneal dialysis among patients admitted over the weekend

Variables	Coefficient	Linearized standard error	t-value	P-value	95% CI (lower limit)	95% CI (upper limit)
Peritoneal dialysis						
Weekend admission	1.754	0.341	2.89	<0.001	1.199	2.566
Age (in years)	0.99	0.006	-1.61	0.12	0.979	1.002
Female	1.001	0.173	0.00	1.00	0.713	1.405
Race (White as reference)	1					
Black	0.559	0.124	-2.61	0.01	0.362	0.865
Hispanic	0.683	0.191	-1.36	0.17	0.394	1.183
Asian or Pacific Islander	1.015	0.443	0.03	0.97	0.431	2.39
Native American	0.704	0.715	-0.35	0.73	0.096	5.157
Other	1.373	0.595	0.73	0.46	0.588	3.209
Median household income for patients' ZIP code, US$ (1-45,999 as reference)	1					
46,000-58,999	1.401	0.33	1.43	0.15	0.883	2.222
59,000-78,999	1.362	0.365	1.15	0.25	0.805	2.305
79,000 or more	1.663	0.527	1.61	0.11	0.894	3.094
Charlson Comorbidity Index	0.952	0.046	-1.03	0.30	0.866	1.046
Hospital region (Northeast as reference)	1					
Midwest	1.392	0.441	1.04	0.30	0.748	2.591
South	1.413	0.47	1.04	0.30	0.737	2.712
West	0.985	0.323	-0.05	0.963	0.518	1.874
Hospital location/teaching status	1.373	0.305	1.43	0.15	0.888	2.123
Hospital bed size (small as reference)	1					
Medium	0.976	0.307	-0.08	0.94	0.527	1.808
Large	1.307	0.361	0.97	0.33	0.76	2.247

About 62% of weekday admissions (709,518) received in-hospital HD, including CRRT (which is defined as continuous urine filtration for more than 18 hours per day) during the same admission. Of the patients admitted on weekends, 60% received HD (p = 0.0000). Patients admitted on weekends had lower odds of receiving HD compared to patients admitted on weekdays (OR: 0.90; 95% CI: 0.88-0.92; p = 0.000). Higher odds of HD on weekends were independently linked with the Black race and higher CCI scores. These probabilities were not related to age, female sex, or admission to a hospital located in the west. The mortality rate among patients receiving HD in the study was 4.2% (Table 4).

**Table 4:** Adjusted odds of hemodialysis among patients admitted over the weekend

Variables	Coefficient	Linearized standard error	t-value	P-value	95% CI (lower limit)	95% CI (upper limit)
Hemodialysis						
Weekend admission	0.899	0.01	-9.26	<0.001	0.879	0.919
Age (in years)	0.998	0	-3.50	<0.001	0.998	0.999
Female	0.955	0.01	-4.38	<0.001	0.936	0.975
Race (White as reference)	1					
Black	1.45	0.035	15.42	<0.001	1.383	1.52
Hispanic	1.229	0.064	3.97	<0.001	1.11	1.361
Asian or Pacific Islander	1.218	0.049	4.95	<0.001	1.127	1.318
Native American	1.283	0.101	3.17	0.002	1.1	1.497
Other	1.141	0.079	1.90	0.058	0.996	1.307
Median household income for patients' ZIP code, US$ (1-45,999 as reference)	1					
46,000-58,999	0.899	0.024	-3.95	<0.001	0.852	0.948
59,000-78,999	0.877	0.025	-4.54	<0.001	0.828	0.928
79,000 or more	0.859	0.029	-4.44	<0.001	0.803	0.919
Charlson Comorbidity Index	1.136	0.006	25.81	<0.001	1.125	1.147
Hospital region (Northeast as reference)	1					
Midwest	1.028	0.062	0.46	0.645	0.913	1.157
South	0.906	0.055	-1.64	0.101	0.805	1.019
West	0.998	0.065	-0.03	0.979	0.878	1.135
Hospital location/teaching status	0.896	0.03	-3.32	0.001	0.84	0.956
Hospital bed size (small as reference)	1					
Medium	0.964	0.054	-0.66	0.511	0.864	1.076
Large	0.868	0.045	-2.76	0.006	0.784	0.96

The prevalence of renal TP in the study population was 0.01% (11,444). On univariate analysis, patients admitted on weekends had a greater likelihood of receiving a kidney transplant (OR: 1.32; 95% CI: 1.20-1.45; p = 0.000). On multivariable regression analysis, similar results were observed after adjusting for patient and hospital-level variables (OR: 1.34; 95% CI: 1.21-1.48; p = 0.000). Higher income in the patient's ZIP code, admission to urban teaching hospitals, or institutions with higher bed numbers were all associated with an increased likelihood of renal TP. Female gender, Black and Asian/Pacific Islanders, a higher CCI, and being older were all related to a lower chance of receiving a renal transplant on weekends (Table 5).

**Table 5:** Adjusted odds of renal transplantation among patients admitted over the weekend

Variables	Coefficient	Linearized standard error	t-value	P-value	95% CI (lower limit)	95% CI (upper limit)
Renal transplantation						
Weekend admission	1.343	0.068	5.82	<0.001	1.216	1.483
Age (in years)	0.978	0.002	-14.25	<0.001	0.975	0.981
Female	0.842	0.04	-3.59	<0.001	0.767	0.925
Race (White as reference)	1					
Black	0.946	0.082	-0.64	0.52	0.799	1.121
Hispanic	1.026	0.118	0.23	0.82	0.819	1.286
Asian or Pacific Islander	1.454	0.211	2.58	0.010	1.094	1.934
Native American	0.679	0.225	-1.17	0.24	0.355	1.299
Other(	1.075	0.228	0.34	0.73	0.71	1.628
Median household income for patients' ZIP code, US$ 1-45,999 as reference)	1					
46,000-58,999	1.196	0.087	2.47	0.01	1.038	1.378
59,000-78,999	1.431	0.124	4.13	<0.001	1.207	1.697
79,000 or more	1.587	0.166	4.40	<0.001	1.292	1.949
Charlson Comorbidity Index	0.71	0.014	-17.86	<0.001	0.684	0.737
Hospital region (Northeast as reference)	1					
Midwest	0.954	0.185	-0.24	0.81	0.652	1.395
South	1.041	0.199	0.21	0.83	0.716	1.514
West	1.119	0.267	0.47	0.64	0.702	1.786
Hospital location/teaching status	37.647	21.19	6.45	<0.001	12.488	113.495
Hospital bed size (small as reference)	1					
Medium	2.284	1.284	1.47	0.14	0.759	6.875
Large	7.263	3.61	3.99	<0.001	2.741	19.245

The overall average time to any RRT was 1.6 days. Patients admitted on weekends had a 1.8-day average time to RRT, compared to 1.5 days on weekdays. While controlling for the potential patient and hospital-level variables, patients hospitalized on weekends had a 29% lower chance of receiving any RRT within 24 hours of admission (OR: 0.71; 95% CI: 0.70-0.73; p = 0.000).

The total length of hospital stay and total hospitalization charges

The average LOS for patients in the study was 6.2 days (95% CI: 6.54-6.71). The average LOS for all weekend admissions was 6.64, which was comparable to the LOS for weekday admissions (6.62). Patients admitted over the weekend who received early RRT (within 24 hours of admission) had a shorter mean LOS (5.7 days; 95% CI: 5.63-5.84) than those who did not (7.2 days; 95% CI: 0.70-0.73). Early RRT was independently linked with shorter LOS in multivariable linear regression analysis (p = 0.000). Longer hospital stay was also associated with older age, greater median income in the patient's ZIP code, and a higher CCI score.

In the study population, the average total hospitalization charge was $83,358 (95% CI: $80,522-$86,194). When compared to patients treated on weekdays (mean: $82,879; 95% CI: $80,103-$85,655), those admitted on weekends had an average hospital bill of $84,982 (95% CI: $81,645-$88,319). After correcting for potential confounders in a linear regression model, patients admitted on weekends paid $1,451 higher in mean total hospitalization expenditures when compared to patients admitted on weekdays (p = 0.07). Higher mean total hospital charges were independently associated with higher median income in patients' ZIP codes, admission into urban teaching hospitals, and a higher CCI. Patients who underwent early RRT had a mean total hospital charge of $77,035 (95% CI: $74,121-$79,950) compared with those who did not (mean: $87,888; 95% CI: $84,807-$90,924). After controlling for patient and hospital-level variables, early RRT was found to be related to lower overall average hospitalization charges in a regression analysis. Female gender, Native American race, and hospital admissions in the Midwest were all related to lower average overall hospital expenses.

## Discussion

The major findings in this study were as follows: (1) patients admitted on weekdays had 8% higher odds of in-hospital mortality compared to patients admitted on weekends. (2) The odds of receiving an RRT over the weekend were 9% lower than on weekdays. (3) The overall rate of PD within the study population was less than 1% and more likely to occur during a weekend admission. (4) HD (including CRRT) was the most common RRT received by the study population (62%), and on weekends, comparable proportions of HD sessions were performed as on weekdays. (5) Patients admitted over the weekend had 10% lower odds of receiving HD. (6) Patients admitted over the weekend had higher renal transplant rates compared to weekday admissions (1.2% vs. 0.9%, respectively), and being female, older, Black, Asian, or Pacific Islander, or having a higher CCI score reduced the odds of receiving a renal transplant. (7) Patients admitted on weekends, on average, wait longer to receive any RRT than patients admitted on weekdays, but have a similar LOS. (8) At discharge, patients admitted on weekends have higher mean total hospitalization expenditures.

ESRD is the ninth leading cause of death in the US [1]. A total of 62.9% of deaths from ESRD occur within the hospital [12]. Variables such as age, race, type, and day of admission, expected primary payer, type of hospital control, and severity of comorbidities have been identified as predictors of in-hospital mortality [13]. The index study identified weekend admission, older age, higher CCI score, and admission into medium or large hospitals as important determinants of in-hospital mortality in this patient population. This could be explained in part by the notion that the patients admitted on weekends are at a higher risk, since they are admitted as emergencies, than the group admitted on weekdays, and the observed weekend mortality effect is mostly due to this risk imbalance [14]. Inadequate staffing on weekends, delay in the commencement of procedural care and interventions, and a high burnout rate have been identified as important determinants of care and in-hospital mortality in ESRD [15]. Other studies in the US have also found a comparable elevated risk of death linked to weekend acute hospitalizations for acute renal injury [16,17].

A mortality rate of up to 15-20% and a five-year survival rate of under 50% have been reported for patients on dialysis [18]. Of the patients receiving HD in this study, 4.2% died during hospitalization. As seen in this study, a similar proportion of HD sessions may be scheduled on weekends compared to weekdays for a variety of reasons, including HD treatments can be time-consuming and may last several hours, and patients (especially those on CRRT) may prefer to have them on weekends when they have more time to rest and recover; some patients may be unable to schedule sessions during the week due to work or other commitments; and patients may be able to schedule sessions on weekends because some dialysis centers have more availability on weekends. Also, some dialysis centers may be more crowded during the week, so some patients are scheduled for treatments on weekends to ensure they receive optimal care. HD normally requires patients to adhere to a rigid treatment regimen that includes thrice-weekly dialysis on either Monday-Wednesday-Friday (MWF) or Tuesday-Thursday-Saturday (TTS). Electrolytes, fluid, and different uremic toxins accumulate during the periods between dialysis sessions, contributing to an increased risk of mortality. As a result, the MWF or TTS dialysis schedule may place patients at greater risk of death on some days. Patients on the MWF schedule, in particular, may be at a higher risk of death on Monday, and those on a TTS schedule may be at a higher risk on Tuesday because these days follow the longest periods without the benefit of dialysis. Research examining schedule-specific mortality and the factors that influence it has discovered that patients on the TTS schedule had higher mortality on Saturdays while those on the MWF schedule had higher mortality on Fridays [19]. This suggests the possible influence of practice patterns on weekend mortality.

There could be a number of explanations for why a higher proportion of patients admitted on weekends in this study received kidney transplants than weekday admissions. One possibility is that there are fewer elective surgeries scheduled on weekends, so there may be more availability for transplant surgeries. Another reason could be that transplant surgeons and other medical staff may have more time to devote to transplant surgeries on weekends, since they may have fewer other commitments. It is also possible that there are logistical reasons that make it easier to schedule transplant surgeries on weekends. However, without more information, it is difficult to determine why a higher proportion of patients received renal transplants on weekends in this study. African Americans are 3.5 times as likely than Whites to have renal failure, are more likely to require dialysis, and account for more than 33% of individuals on the waiting list for a renal transplant [20]. Being female, older, and having higher CCI scores have all been associated with a lower likelihood of receiving a transplant in previous reports [21,22]. The findings of the index study corroborate these reports.

Patients admitted on weekends in the index study had a comparable LOS to patients admitted on weekdays. There could be several reasons why the LOS might be similar for patients admitted on weekends and weekdays. One possibility is that the severity of the patients’ conditions and the types of treatments required are similar regardless of the day of admission. Additionally, the availability of certain medical resources and staffing levels might be consistent across weekdays and weekends in many hospitals, leading to similar LOS for patients. It is also plausible that hospital policies or protocols are in place to ensure that patients receive a certain level of care and treatment regardless of the day of admission [23,24].

Limitations

This study had some limitations. Firstly, biases inherent in retrospective studies apply to NIS database studies. For example, the NIS employs ICD-10-CM/PCS codes to define diagnoses and hospitalization stays, and as a result, coding inaccuracies are a possibility. Secondly, the majority of ICD-10-CM/PCS codes utilized by the database do not indicate the severity of an illness. We were unable to ascertain if the severity of the underlying disease had an impact on the outcomes of ESRD patients admitted on the weekends. Thirdly, because this study represents data on hospitalizations for ESRD rather than data on specific patients, anyone who was hospitalized more than once with the same primary diagnosis would be counted multiple times. It was not possible to assess the cause of mortality or the impact of the conventional thrice-weekly dialysis schedules on mortality during the weekend since the weekend and mortality variables in NIS are dichotomous variables. Finally, the NIS database does not contain information on medications taken while hospitalized or test results that would indicate the severity of an underlying disease. The NIS database continues to be a trustworthy resource and is frequently used in research despite its limitations.

## Conclusions

Patients with ESRD admitted on weekends had lower overall rates of RRT and had to wait longer for RRT than those admitted on weekdays. However, the average LOS for ESRD patients does not appear to differ significantly depending on the day of admission. This suggests that there may be other factors at play that contribute to the observed differences in patient outcomes between weekend and weekday admissions. Overall, the weekend effect highlights the need for further research to understand the underlying causes of this phenomenon and to identify strategies to mitigate its negative consequences for patients with ESRD.

## Appendices

**Table 6:** Procedural ICD-10-CM/PCS codes used in the study ICD-10-CM/PCS: International Classification of Diseases, Tenth Revision, Clinical Modification/Procedure Coding System.

Variable	Procedural ICD-10-CM/PCS code
Performance of urinary filtration, continuous, greater than 18 hours per day	5A1D90Z
Irrigation of peritoneal cavity using the irrigating substance, percutaneous approach	3E1M38Z
Transplantation of left kidney, zooplastic, open approach	0TY10Z2
Transplantation of left kidney, allogeneic, open approach	0TY10Z1
Transplantation of left kidney, allogeneic, open approach	0TY10Z0
Transplantation of right kidney, allogeneic, open approach	0TY00Z0
Transplantation of right kidney, syngeneic, open approach	0TY00Z1
Transplantation of right kidney, zooplastic, open approach	0TY00Z2
Performance of urinary filtration, intermittent, less than 6 hours per day	5A1D70Z
Performance of urinary filtration, prolonged intermittent, 6-18 hours per day	5A1D80Z

**Table 7:** Diagnostic ICD-10-CM/PCS codes used in the study ICD-10-CM/PCS - International Classification of Diseases, Tenth Revision, Clinical Modification/Procedure Coding System.

Variable	Diagnostic ICD-10-CM/PCS Code
End-stage renal disease	N186
Hypertensive chronic kidney disease with stage 5 chronic kidney disease or end-stage renal disease	I120
Hypertensive heart and chronic kidney disease without heart failure, with stage 5 chronic kidney disease, or end-stage renal disease	I1311
Hypertensive heart and chronic kidney disease with heart failure and with stage 5 chronic kidney disease, or end-stage renal disease	I132
Dependence on renal dialysis	Z992
Patient's noncompliance with renal dialysis	Z9115
Encounter for fitting and adjustment of extracorporeal dialysis catheter	Z4901
Encounter for fitting and adjustment of peritoneal dialysis catheter	Z4902
Encounter for adequacy testing for hemodialysis	Z4931
Encounter for adequacy testing for peritoneal dialysis	Z4932
Cloudy (hemodialysis) (peritoneal) dialysis effluent	R880
Unspecified complication of foreign body accidentally left in body following kidney dialysis, initial encounter	T81512A
Unspecified complication of foreign body accidentally left in body following kidney dialysis, subsequent encounter	T81512D
Unspecified complication of foreign body accidentally left in body following kidney dialysis, sequela	T81512S
Obstruction due to a foreign body accidentally left in the body following kidney dialysis, initial encounter	T81522A
Obstruction due to a foreign body accidentally left in the body following kidney dialysis, subsequent encounter	T81522D
Obstruction due to a foreign body accidentally left in the body following kidney dialysis, sequela	T81522S
Perforation due to a foreign body accidentally left in the body following kidney dialysis, initial encounter	T81532A
Perforation due to a foreign body accidentally left in the body following kidney dialysis, subsequent encounter	T81532D
Perforation due to a foreign body accidentally left in the body following kidney dialysis, sequela	T81532S
Other complications of foreign body accidentally left in the body following kidney dialysis, initial encounter	T81592A
Other complications of foreign body accidentally left in the body following kidney dialysis, subsequent encounter	T81592D
Other complications of foreign body accidentally left in the body following kidney dialysis, sequela	T81592S
Breakdown (mechanical) of vascular dialysis catheter	T8241
Breakdown (mechanical) of vascular dialysis catheter, initial encounter	T8241XA
Breakdown (mechanical) of vascular dialysis catheter, subsequent encounter	T8241XD
Breakdown (mechanical) of vascular dialysis catheter, sequela	T8241XS
Displacement of vascular dialysis catheter	T8242
Displacement of vascular dialysis catheter, initial encounter	T8242XA
Displacement of vascular dialysis catheter, subsequent encounter	T8242XD
Displacement of vascular dialysis catheter, sequela	T8242XS
Leakage of vascular dialysis catheter	T8243
Leakage of vascular dialysis catheter, initial encounter	T8243XA
Leakage of vascular dialysis catheter, subsequent encounter	T8243XD
Leakage of vascular dialysis catheter, sequela	T8243XS
Other complications of vascular dialysis catheter	T8249
Other complications of vascular dialysis catheter, initial encounter	T8249XA
Other complications of vascular dialysis catheter, subsequent encounter	T8249XD
Other complications of vascular dialysis catheter, sequela	T8249XS
Breakdown (mechanical) of intraperitoneal dialysis catheter, initial encounter	T85611A
Breakdown (mechanical) of intraperitoneal dialysis catheter, subsequent encounter	T85611D
Breakdown (mechanical) of intraperitoneal dialysis catheter, sequela	T85611S
Displacement of intraperitoneal dialysis catheter, initial encounter	T85621A
Displacement of intraperitoneal dialysis catheter, subsequent encounter	T85621D
Displacement of intraperitoneal dialysis catheter, sequela	T85621S
Leakage of intraperitoneal dialysis catheter, initial encounter	T85631A
Leakage of intraperitoneal dialysis catheter, subsequent encounter	T85631D
Leakage of intraperitoneal dialysis catheter, sequela	T85631S
Other mechanical complications of intraperitoneal dialysis catheter, initial encounter	T85691A
Other mechanical complications of intraperitoneal dialysis catheter, subsequent encounter	T85691D
Other mechanical complications of intraperitoneal dialysis catheter, sequela	T85691S
Infection and inflammatory reaction due to peritoneal dialysis catheter, initial encounter	T8571XA
Infection and inflammatory reaction due to peritoneal dialysis catheter, subsequent encounter	T8571XD
Infection and inflammatory reaction due to peritoneal dialysis catheter, sequela	T8571XS
Failure of sterile precautions during kidney dialysis and other perfusion	Y622
Kidney dialysis as the cause of abnormal reaction of the patient, or of later complication, without mention of misadventure at the time of the procedure	Y841
Complications of kidney transplant	T861
Unspecified complications of kidney transplant	T8610
Kidney transplant rejection	T8611
Kidney transplant failure	T8612
Kidney transplant rejection	T8613
Other complications of kidney transplant	T8619
Encounter for aftercare following kidney transplant	Z4822
Kidney transplant status	Z940
